# Effects of prehospital epinephrine during out-of-hospital cardiac arrest with initial non-shockable rhythm: an observational cohort study

**DOI:** 10.1186/cc12872

**Published:** 2013-09-03

**Authors:** Yoshikazu Goto, Tetsuo Maeda, Yumiko Nakatsu Goto

**Affiliations:** 1 Section of Emergency Medicine, Kanazawa University Hospital, 13-1 Takaramachi, Kanazawa 920-8641, Japan; 2 Department of Cardiology, Yawata Medical Center, 12-7 I Yawata, Komatsu 923-8551, Japan

## Abstract

**Introduction:**

Few clinical trials have provided evidence that epinephrine administration after out-of-hospital cardiac arrest (OHCA) improves long-term survival. Here we determined whether prehospital epinephrine administration would improve 1-month survival in OHCA patients.

**Methods:**

We analyzed the data of 209,577 OHCA patients; the data were prospectively collected in a nationwide Utstein-style Japanese database between 2009 and 2010. Patients were divided into the initial shockable rhythm (*n *= 15,492) and initial non-shockable rhythm (*n *= 194,085) cohorts. The endpoints were prehospital return of spontaneous circulation (ROSC), 1-month survival, and 1-month favorable neurological outcomes (cerebral performance category scale, category 1 or 2) after OHCA. We defined epinephrine administration time as the time from the start of cardiopulmonary resuscitation (CPR) by emergency medical services personnel to the first epinephrine administration.

**Results:**

In the initial shockable rhythm cohort, the ratios of prehospital ROSC, 1-month survival, and 1-month favorable neurological outcomes in the non-epinephrine group were significantly higher than those in the epinephrine group (27.7% vs. 22.8%, 27.0% vs. 15.4%, and 18.6% vs. 7.0%, respectively; all *P *< 0.001). However, in the initial non-shockable rhythm cohort, the ratios of prehospital ROSC and 1-month survival in the epinephrine group were significantly higher than those in the non-epinephrine group (18.7% vs. 3.0% and 3.9% vs. 2.2%, respectively; all *P *< 0.001) and there was no significant difference between the epinephrine and non-epinephrine groups for 1-month favorable neurological outcomes (*P = *0.62). Prehospital epinephrine administration for OHCA patients with initial non-shockable rhythms was independently associated with prehospital ROSC (adjusted odds ratio [aOR], 8.83, 6.18, 4.32; 95% confidence interval [CI], 8.01-9.73, 5.82-6.56, 3.98-4.69; for epinephrine administration times ≤9 min, 10-19 min, and ≥20 min, respectively), with improved 1-month survival when epinephrine administration time was <20 min (aOR, 1.78, 1.29; 95% CI, 1.50-2.10, 1.17-1.43; for epinephrine administration times ≤9 min and 10-19 min, respectively), and with deteriorated 1-month favorable neurological outcomes (aOR, 0.63, 0.49; 95% CI, 0.48-0.80, 0.32-0.71; for epinephrine administration times 10-19 min and ≥20 min, respectively).

**Conclusions:**

Prehospital epinephrine administration for OHCA patients with initial nonshockable rhythms was independently associated with achievement of prehospital ROSC and had association with improved 1-month survival when epinephrine administration time was <20 min.

## Introduction

Out-of-hospital cardiac arrest (OHCA) is an increasing public health concern in industrial countries with aging populations [1-4]. Survival after OHCA has not significantly improved in almost three decades, despite enormous research and the development of novel drugs and devices [5]. In Japan, more than 100,000 OHCA cases occur every year [1,2,6,7]. Nationwide improvements in favorable neurological outcomes following cardiac arrest have been observed after connecting the links in the "chain of survival" [1,8]. Epinephrine has been a cornerstone of cardiac resuscitation therapy and advanced cardiac life support since the 1960s [9]. Epinephrine increases aortic blood pressure and coronary perfusion pressure during chest compression in animals [10,11]. In humans, high-dose epinephrine has been shown to raise coronary perfusion pressure and may improve rates of return to spontaneous circulation (ROSC) [12]. The most recent advanced life support guidelines for the treatment of cardiac arrest due to ventricular fibrillation (VF) recommend the administration of either epinephrine or vasopressin as the first drug after defibrillation [13]. Also, epinephrine is the recommended first-line drug for the resuscitation of patients with both asystole and pulseless electrical activity (PEA) [13]. However, there is little evidence from clinical trials that epinephrine administration after OHCA improves long-term survival [6,14,15]. Increased myocardial dysfunction [16,17] and disturbed cerebral microcirculation [18] after epinephrine administration may contribute importantly to the poor long-term outcomes.

A recent randomized controlled trial (RCT) [14] showed that patients with an initial nonshockable rhythm had higher ratios of short-term survival when intravenous therapy was administered, whereas no differences in outcomes were found for patients with a shockable rhythm. Moreover, as some patients do recover after administration of epinephrine, there are subsets of patients for whom epinephrine administration is in fact beneficial [9]. Therefore, the first objective of the present study was to examine whether initial cardiac rhythm should be considered a key factor for predicting survival and favorable neurological outcomes at one month after OHCA. The second objective was to determine whether pre-hospital epinephrine administration improves one-month survival in patients who experienced OHCA with initial nonshockable rhythms.

## Materials and methods

### Study design and data source

The present investigation was a nationwide population-based observational study of all adult patients (ages 18 years and older) in Japan for whom resuscitation was attempted after OHCA from 1 January 2009 to 31 December 2010. *Cardiac arrest *was defined as the cessation of cardiac mechanical activities as confirmed by the absence of signs of circulation [1]. The cause of arrest was presumed to be cardiac unless evidence suggested external causes (trauma, hanging, drowning, drug overdose and asphyxia), respiratory diseases, cerebrovascular diseases, malignant tumors or any other noncardiac cause. Attribution of noncardiac or cardiac cause was made by the physicians in charge in collaboration with the emergency medical services (EMS) personnel. This study was approved by the Ethical Committee of Kanazawa University. The requirement for written informed consent was waived.

### Emergency medical services system in Japan

Japan has approximately 127 million residents in an area of 378,000 km^2^, approximately two-thirds of which is uninhabited mountainous terrain [1,19]. Details of the Japanese EMS system have been described previously [1,2,6,7,19-21]. Briefly, municipal governments provide EMS through about 800 fire stations with dispatch centers. The Fire and Disaster Management Agency (FDMA) of Japan supervises the nationwide EMS system [1,6,7,19,20], while each local EMS system is operated by the local fire station. Generally, an ambulance crew includes three EMS staff members, including at least one emergency lifesaving technician (ELST) [1]. ELSTs are allowed to use several resuscitation methods, including semi-automated external defibrillators, insertion of a supraglottic airway device (laryngeal mask airway, laryngeal tube, and esophageal-tracheal twin-lumen airway device), insertion of a peripheral intravenous line and administration of Ringer lactate solution [1]. Since July 2004, only specially trained ELSTs have been permitted to insert a tracheal tube, and, since April 2006, they have been permitted to administer intravenous epinephrine in the field under online physician instruction [1,2,6,7]. Since October 2006, all EMS providers have been permitted to perform cardiopulmonary resuscitation (CPR) according to the Japanese CPR guidelines [21], which are based on the 2005 American Heart Association guidelines [4]. As EMS personnel in Japan are legally prohibited from terminating resuscitation in the field, most OHCA patients undergo CPR by EMS providers and are transported to hospitals, except in cases where fatality is certain [1]. Epinephrine use is implemented according to the FDMA resuscitation guidelines for ELST [21,22]. The guidelines allow ELSTs to attempt intravenous access only twice, and each attempt must take no longer than 90 seconds. The allowable dosage of epinephrine is 1 mg per attempt, and repeated doses may be administered under a physician's instruction.

### Data collection and quality control

The FDMA launched a prospective, population-based observational study involving all OHCA patients who received EMS in Japan [1]. EMS personnel at each center recorded data for OHCA patients with the cooperation of the physician in charge using an Utstein-style template [23]. All data were transferred and stored in the nationwide database developed by the FDMA for public use. We analyzed this database with the permission of the FDMA, which provided all the anonymous data to our research group.

The main items included in the data set were as follows: sex, age, causes of arrest (presumed cardiac origin or not), bystander witness status, bystander CPR with or without automated external defibrillator use, initial identified cardiac rhythm, bystander category (that is, if there was a bystander, whether the bystander was a layperson or EMS personnel), whether epinephrine was administered, whether advanced airway management techniques (including endotracheal tube, laryngeal mask airway, and esophageal-tracheal tube) were used, whether ROSC was attained before arrival at the hospital, time of the emergency call, time of vehicle arrival at the scene, time of ROSC, time of vehicle arrival at the hospital, time of epinephrine administration, one-month survival and neurological outcome at one month after cardiac arrest. The neurological outcome was defined using the Cerebral Performance Categories (CPC) scale score: category 1, good cerebral performance; category 2, moderate cerebral disability; category 3, severe cerebral disability; category 4, coma or vegetative state; and category 5, death [23]. CPC categorization was determined by the physician in charge. The call-to-response time was calculated as the time from the emergency call to the time of vehicle arrival at the scene. The call-to-hospital arrival time was calculated as the time from the emergency call to the time of vehicle arrival at the hospital.

### Endpoints

The primary study endpoint was survival at one month. The secondary endpoints were ROSC before arrival at the hospital and survival at one month with favorable neurological outcome (defined as a CPC score of 1 or 2) [23].

### Statistical analysis

Kolmogorov-Smirnov and Lilliefors tests were performed to evaluate the distributions of continuous variables, and we found that all continuous variables were not normally distributed (all *P *< 0.01). Therefore, the Wilcoxon and Kruskal-Wallis tests for continuous variables, and the χ^2 ^test for categorical variables, were performed to compare the characteristics or outcomes between the cohorts in each initial cardiac rhythm. Multivariate logistic regression analyses, including 12 variables, were performed to assess the factors contributing to one-month survival and one-month CPC score of 1 or 2 for all eligible patients. The 12 selected variables included year, age, gender, witnessed arrest, bystander CPR, arrest presumed cause, initial cardiac rhythm, prehospital shock delivery, advanced airway management, call-to-response time, call-to-hospital arrival time and pre-hospital epinephrine administration for the model as an independent variable. These analyzing models yielded concordance statistics of 0.81 for one-month survival and 0.89 for one-month CPC scores of 1 or 2, respectively, which indicated good discrimination.

Moreover, multivariate logistic analysis including 11 variables was used to determine the impact of prehospital epinephrine administration for pre-hospital ROSC, one-month survival, and one-month CPC score of 1 or 2 in each initial cardiac rhythm. The 11 selected variables included year, age, gender, witnessed arrest, bystander CPR, arrest presumed cause, initial cardiac rhythm, prehospital shock delivery, advanced airway management, call-to-response time and pre-hospital epinephrine administration for the model as an independent variable.

In these multivariate logistic regression analyses for outcomes, we classified the following two continuous variables into four categories, respectively: age (≤39 years, 40 to 59 years, 60 to 79 years and ≥80 years) and call-to-response time (≤4 minutes, 5 to 9 minutes, 10 to 14 minutes and ≥15 minutes). We defined epinephrine administration time as the time interval from the start of CPR by EMS personnel to the first epinephrine administration. In order to associate the epinephrine administration time with whether epinephrine was received, we classified prehospital epinephrine administration variables into four categories, No, Yes (≤9 minutes), Yes (10 to 19 minutes) and Yes (≥20 minutes), where the figures in parentheses are the epinephrine administration times.

Continuous variables are expressed as means and standard deviations. Categorical variables are expressed as percentages. As an estimate of effect size and variability, we report odds ratios (ORs) with 95% confidence intervals (CIs). All statistical analyses were performed using the JMP statistical software package version 10 (SAS Institute Inc, Cary, NC, USA). All tests were two-tailed, and *P *< 0.05 was considered statistically significant.

## Results

**Figure 1:**
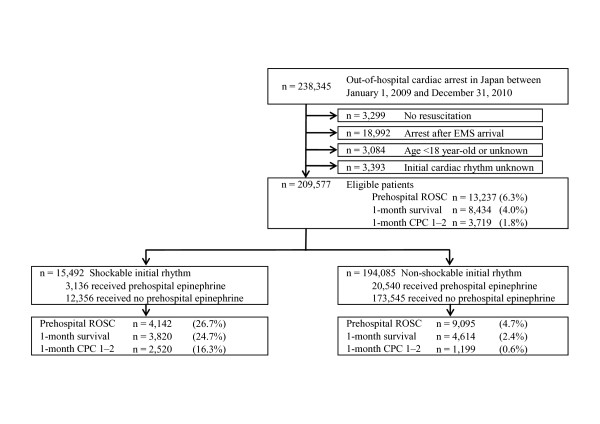
**Study profile showing participant selection**. CPC, Cerebral Performance Categories; EMS, emergency medical services; ROSC, return of spontaneous circulation.

During the two-year study period, details of 238,345 patients were documented in the database. We considered 209,577 patients (87.9%) eligible for enrollment into this study. Figure 1 depicts the inclusion and exclusion criteria for participants in the present study. Overall pre-hospital ROSC, one-month survival and one-month CPC score of 1 or 2 were 6.3% (*n *= 13,237), 4.0% (*n *= 8,434) and 1.8% (*n *=3,419), respectively. Of these arrests, 15,492 (7.4%) had an initial shockable rhythm and 194,085 (92.6%) had an initial nonshockable rhythm. The ratios of both short-term and long-term outcomes in the initial shockable rhythm cohort were significantly higher than those in the initial nonshockable rhythm cohort (26.7% vs. 4.7% for prehospital ROSC, 24.7% vs. 2.4% for 1-month survival and 16.3% vs. 0.6% for one-month CPC score of 1 or 2; all *P *< 0.001). Table 1 shows the baseline characteristics of study patients and the results of multivariate logistic regression analyses for 12 prehospital factors in predicting one-month outcomes after OHCA. Initial shockable rhythm was an independent contributing factor in both survival (adjusted OR, 4.59; 95% CI, 4.13 to 5.10) and CPC score of 1 or 2 (adjusted OR, 5.46; 95% CI, 4.67 to 6.42) at one month after OHCA with the highest adjusted OR among variables. Although prehospital epinephrine administration had no significant factors regarding one-month survival, it was independently associated with deteriorated neurological outcomes at one month.

**Table 1:** Characteristics of patients and contributing factors to one-month outcomes after out-of-hospital cardiac arrest^a^

Characteristics	All patients		Adjusted OR (95% CI)			
			
	(*N *= 209,577)		One-month survival		One-month CPC score 1 or 2	
Year 2010	107,952	(51.5)	1.06	(1.01 to 1.11)	1.07	(1.00 to 1.15)
Mean age^b ^(yr)	74.0	(± 16.1)	0.24	(0.21 to 0.28)	0.97	(0.97 to 0.98)
Male	120,784	(57.6)	0.99	(0.94 to 1.04)	1.06	(0.97 to 1.15)
Witnessed arrest	74,788	(35.7)	3.34	(3.17 to 3.52)	3.31	(3.04 to 3.61)
Bystander CPR	95,672	(45.7)	1.14	(1.09 to 1.19)	1.31	(1.23 to 1.42)
Presumed cardiac cause	118,908	(56.7)	0.85	(0.81 to 0.90)	1.59	(1.45 to 1.75)
Shockable initial cardiac rhythm	15,492	(7.4)	4.59	(4.13 to 5.10)	5.46	(4.67 to 6.42)
Prehospital actual shock delivery	21,653	(10.3)	1.97	(1.77 to 2.18)	3.00	(2.55 to 3.53)
Use of advanced airway management	90,892	(43.3)	0.83	(0.79 to 0.87)	0.48	(0.44 to 0.52)
Call-to-response time,^b ^min	7.6	(± 3.8)	0.003	(0.001 to 0.004)	0.87	(0.86 to 0.88)
Call-to-hospital arrival time,^b ^min	32.8	(± 12.1)	0.45	(0.35 to 0.59)	1.00	(0.99 to 1.00)
Prehospital epinephrine administration	23,676	(11.3)	0.98	(0.92 to 1.05)	0.47	(0.42 to 0.54)

^a^CPC, Cerebral Performance Categories; CPR, cardiopulmonary resuscitation; OR, odds ratio; CI, confidence interval. Values are reported either as *n *(%) or mean ± standard deviation. Values were missing for 492 to 590 individuals across time variables. ^b^Adjusted odds ratios are reported for unit odds.

Table 2 shows baseline characteristics of study patients according to the initial cardiac rhythm and the presence of prehospital epinephrine administration. Among patients who received prehospital epinephrine, call-to-response time and epinephrine administration time in the initial shockable rhythm cohort were significantly shorter than in the initial nonshockable rhythm cohort (all *P *< 0.0001). Table 3 shows both short-term and long-term outcomes according to the initial cardiac rhythm and the presence of prehospital epinephrine administration. In the initial shockable rhythm cohort, the ratios of both short-term and long-term outcomes in the nonepinephrine group were significantly higher than those in the epinephrine group (27.7% vs. 22.8% for prehospital ROSC, 27.0% vs. 15.4% for one-month survival and 18.6% vs. 7.0% for one-month CPC score of 1 or 2; all *P *< 0.001). However, in the initial nonshockable rhythm cohort, the ratios of pre-hospital ROSC and one-month survival in the epinephrine group were significantly higher than those in the nonepinephrine group (18.7% vs. 3.0% and 3.9% vs. 2.2%, respectively; all *P *< 0.001). No significant difference between the epinephrine and nonepinephrine groups for one-month CPC score of 1 or 2 was found in the initial nonshockable rhythm cohort (0.59% vs. 0.62%, *P *= 0.605).

**Table 2:** Characteristics of patients according to initial rhythm and pre-hospital epinephrine administration^a^

Characteristics	Initial shockable rhythm				Initial nonshockable rhythm			
	(*N *= 15,492)				(*N *= 194,085)			
		
	Epinephrine		No epinephrine		Epinephrine		No epinephrine	
	(*n *= 3,136)		(*n *= 12,356)		(*n *= 20,540)		(*n *= 173,545)	
Year								
2009	1,423	(45.4)	6,410	(51.9)	9,075	(44.2)	84,717	(48.8)
2010	1,713	(54.6)	5,946	(48.1)	11,465	(55.8)	88,828	(51.2)
Mean age (yr)	66.2	(± 15.0)	66.3	(± 15.4)	74.4	(± 15.0)	74.6	(± 16.1)
Male	2,542	(81.1)	9,411	(76.2)	12,344	(60.1)	96,487	(55.6)
Witnessed arrest	2,237	(71.3)	8,581	(69.5)	10,796	(52.6)	53,174	(30.6)
Bystander CPR	1,610	(51.3)	6,323	(51.2)	9,825	(47.8)	77,914	(44.9)
Presumed cardiac cause	2,794	(89.1)	10,743	(87.0)	11,702	(57.0)	93,669	(54.0)
Initial cardiac rhythm								
Ventricular fibrillation	3,077	(98.1)	12,037	(97.4)	NA		NA	
Pulseless ventricular tachycardia	59	(1.9)	319	(2.6)	NA		NA	
Pulseless electrical activity	NA		NA		7,460	(36.3)	34,153	(19.7)
Asystole	NA		NA		13,080	(63.7)	139,392	(80.3)
Prehospital actual shock delivery	3,003	(95.8)	11,685	(94.6)	1,719	(8.4)	5,246	(3.0)
Use of advanced airway management	2,035	(64.9)	4,695	(38.0)	15,011	(73.1)	69,151	(39.9)
Call-to-response time, min	7.4	(± 3.3)	7.0	(± 3.2)	8.0	(± 4.1)	7.6	(± 3.8)
Epinephrine administration time^b ^(min)	15.5	(± 6.9)	NA		17.0	(± 7.8)	NA	
Total dose of pre-hospital epinephrine (mg)								
1	1,213	(38.7)	NA		8,163	(39.7)	NA	
2	970	(30.9)			6,329	(30.8)		
≥3	953	(30.4)			6,048	(29.4)		

^a^CPR, cardiopulmonary resuscitation; EMS, emergency medical services; NA, not available. Values are reported either as *n *(%) or mean ± standard deviation. Values were missing for 26 to 539 individuals across time variables. ^b^Time from the start of CPR by EMS personnel to the first epinephrine administration.

**Table 3:** Outcomes of patients according to initial rhythm and pre-hospital epinephrine administration^a^

Outcomes	Initial shockable rhythm (*N *= 15,492)					Initial nonshockable rhythm (*N *= 194,085)				
		
	Epinephrine		No epinephrine		*P *value	Epinephrine		No epinephrine		*P *value
	(*n *= 3,136)		(*n *= 12,356)			(*n *= 20,540)		(*n *= 173,545)		
Prehospital ROSC	716	(22.8)	3,426	(27.7)	<0.0001	3,847	(18.7)	5,248	(3.0)	<0.0001
One-month survival	482	(15.4)	3,338	(27.0)	<0.0001	795	(3.9)	3,819	(2.2)	<0.0001
One-month CPC score 1 or 2	219	(7.0)	2,301	(18.6)	<0.0001	121	(0.59)	1,078	(0.62)	0.605

^a^CPC, Cerebral Performance Categories; ROSC, return of spontaneous circulation. Values are reported as *n *(%).

The results of multivariate logistic analyses including 11 variables to determine the factors associated with pre-hospital ROSC, one-month survival and one-month CPC score of 1 or 2 in the initial shockable rhythm cohort are shown in Table 4. Prehospital epinephrine administration, the duration of which was 9 minutes or less, was positively associated only with prehospital ROSC (adjusted OR, 1.45; 95% CI, 1.20 to 1.75). There was no significant difference in one-month survival between no epinephrine and epinephrine administration with an administration time of 9 minutes or less (adjusted OR, 0.95; 95% CI, 0.77 to 1.16). However, a negative association with prehospital epinephrine administration was observed in one-month survival where epinephrine administration time was 10 minutes or longer (adjusted OR, 0.51, 0.33; 95% CI, 0.44 to 0.59, 0.25 to 0.42; for epinephrine administration times of 10 to 19 minutes and 20 minutes or longer, respectively). Moreover, prehospital epinephrine administration was independently associated with deteriorated neurological outcomes at one month (adjusted OR, 0.71, 0.34, 0.21; 95% CI, 0.54 to 0.92, 0.28 to 0.42, 0.14 to 0.31; for epinephrine administration times of 9 minutes or less, 10 to 19 minutes and 20 minutes or longer, respectively).

**Table 4:** Results of multivariate logistic regression analyses for outcomes in the initial shockable rhythm cohort^a^

Variables		Adjusted OR (95% CI)					
		
		Prehospital ROSC		One-month survival		One-month CPC score 1 or 2	
Year							
2009		Reference		Reference		Reference	
2010		1.04	(0.97 to 1.12)	1.07	(0.99 to 1.16)	1.08	(0.99 to 1.19)
Mean age (yr)							
≤39		1.87	(1.58 to 2.21)	4.48	(3.74 to 5.36)	7.58	(6.10 to 9.43)
40 to 59		1.53	(1.36 to 1.73)	3.33	(2.91 to 3.82)	4.73	(3.97 to 5.67)
60 to 79		1.64	(1.47 to 1.82)	2.58	(2.28 to 2.93)	3.26	(2.76 to 3.88)
≥80		Reference		Reference		Reference	
Male		0.87	(0.80 to 0.96)	0.89	(0.80 to 0.98)	0.90	(0.80 to 1.00)
Witnessed arrest		1.96	(1.80 to 2.14)	1.98	(1.80 to 2.17)	2.27	(2.02 to 2.56)
Bystander CPR		1.42	(1.31 to 1.53)	1.43	(1.32 to 1.54)	1.64	(1.49 to 1.80)
Presumed cardiac cause		1.39	(1.23 to 1.57)	2.16	(1.88 to 2.51)	2.50	(2.08 to 3.03)
Initial cardiac rhythm							
Ventricular fibrillation		0.58	(0.44 to 0.76)	0.72	(0.54 to 0.98)	0.75	(0.52 to 1.10)
Pulseless ventricular tachycardia		Reference		Reference		Reference	
Prehospital actual shock delivery		1.95	(1.57 to 2.43)	1.92	(1.52 to 2.46)	2.52	(1.85 to 3.50)
Use of advanced airway management		0.54	(0.50 to 0.58)	0.59	(0.54 to 0.64)	0.43	(0.39 to 0.47)
Call-to-response time (min)							
≤4		3.19	(2.39 to 4.33)	5.88	(4.09 to 8.75)	5.76	(3.72 to 9.40)
5 to 9		2.33	(1.77 to 3.14)	3.89	(2.73 to 5.74)	3.42	(2.23 to 5.54)
10 to 14		1.32	(0.98 to 1.80)	1.95	(1.34 to 2.93)	1.61	(1.02 to 2.67)
≥15		Reference		Reference		Reference	
Pre-hospital epinephrine administration^b^							
No		Reference		Reference		Reference	
Yes	< 9 min	1.45	(1.20 to 1.75)	0.95	(0.77 to 1.16)	0.71	(0.54 to 0.92)
	10 to 19 min	0.88	(0.78 to 1.00)	0.51	(0.44 to 0.59)	0.34	(0.28 to 0.42)
	≥20 min	0.63	(0.52 to 0.77)	0.33	(0.25 to 0.42)	0.21	(0.14 to 0.31)

^a^CI, confidence interval; CPC, Cerebral Performance Categories; CPR, cardiopulmonary resuscitation; OR, odds ratio; ROSC, return of spontaneous circulation. ^b^If pre-hospital epinephrine was received, variables were divided into three categories according to the time from the start of CPR by emergency medical services personnel to the first epinephrine administration (epinephrine administration time).

Table 5 shows the results of multivariate logistic analyses including 11 variables to determine the factors associated with the short-term and long-term outcomes in the initial nonshockable rhythm cohort. Prehospital epinephrine administration was independently associated with prehospital ROSC (adjusted OR, 8.83, 6.18, 4.32; 95% CI, 8.01 to 9.73, 5.82 to 6.56, 3.98 to 4.69; for epinephrine administration times of 9 minutes or less, 10 to 19 minutes and 20 minutes or longer, respectively). Moreover, prehospital epinephrine administration was independently associated with 1-month survival when the first epinephrine administration was performed for less than 20 minutes (adjusted OR, 1.78, 1.29; 95% CI, 1.50 to 2.10, 1.17 to 1.43; for epinephrine administration times of 9 minutes or less and 10 to 19 minutes, respectively). There was no significant difference in one-month CPC score of 1 or 2 between no epinephrine and epinephrine administration when the administration time was 9 minutes or less (adjusted OR, 0.95; 95% CI, 0.62 to 1.37). However, prehospital epinephrine administration was independently associated with deteriorated neurological outcomes at one month when epinephrine administration time was 10 minutes or longer (adjusted OR, 0.63, 0.49; 95% CI, 0.48 to 0.80, 0.32 to 0.71; for epinephrine administration times 10 to 19 minutes and 20 minutes or longer, respectively).

**Table 5:** Results of multivariate logistic regression analyses for outcomes in the initial nonshockable rhythm cohort^a^

Variables		Adjusted OR (95% CI)					
		
		Prehospital ROSC		One-month survival		One-month CPC score 1 or 2	
Year							
2009		Reference		Reference		Reference	
2010		1.04	(0.99 to 1.09)	1.06	(0.99 to 1.12)	1.10	(0.98 to 1.23)
Mean age (yr)							
≤39		1.12	(0.99 to 1.25)	1.53	(1.32 to 1.76)	2.53	(1.91 to 3.29)
40 to 59		1.37	(1.27 to 1.48)	1.57	(1.42 to 1.73)	2.84	(2.37 to 3.41)
60 to 79		1.26	(1.19 to 1.32)	1.50	(1.40 to 1.61)	2.12	(1.85 to 2.44)
≥80		Reference		Reference		Reference	
Male		0.96	(0.92 to 1.01)	0.97	(0.91 to 1.03)	1.11	(0.98 to 1.25)
Witnessed arrest		2.23	(2.12 to 2.34)	2.48	(2.32 to 2.65)	2.37	(2.08 to 2.70)
Bystander CPR		1.09	(1.04 to 1.14)	1.00	(0.94 to 1.06)	0.95	(0.84 to 1.07)
Presumed cardiac cause		0.50	(0.48 to 0.52)	0.66	(0.62 to 0.70)	1.19	(1.06 to 1.34)
Initial cardiac rhythm							
Pulseless electrical activity		3.72	(3.55 to 3.90)	3.61	(3.39 to 3.85)	6.05	(5.31 to 6.92)
Asystole		Reference		Reference		Reference	
Prehospital actual shock delivery		1.22	(1.11 to 1.34)	1.68	(1.49 to 1.89)	2.60	(2.15 to 3.11)
Use of advanced airway management		0.94	(0.90 to 0.98)	0.96	(0.91 to 1.02)	0.55	(0.48 to 0.62)
Call-to-response time (min)							
≤4		1.45	(1.28 to 1.66)	2.90	(2.33 to 3.65)	2.98	(1.95 to 4.81)
5 to 9		1.35	(1.20 to 1.52)	2.38	(1.93 to 2.98)	2.28	(1.52 to 3.64)
10 to 14		1.11	(0.98 to 1.27)	1.56	(1.24 to 1.98)	1.35	(0.87 to 2.22)
≥15		Reference		Reference		Reference	
Pre-hospital epinephrine administration^b^							
No		Reference		Reference		Reference	
Yes	< 9 min	8.83	(8.01 to 9.73)	1.78	(1.50 to 2.10)	0.95	(0.62 to 1.37)
	10 to 19 min	6.18	(5.82 to 6.56)	1.29	(1.17 to 1.43)	0.63	(0.48 to 0.80)
	≥20 min	4.32	(3.98 to 4.69)	0.79	(0.66 to 0.93)	0.49	(0.32 to 0.71)

^a^CI, confidence interval; CPC, Cerebral Performance Categories; CPR, cardiopulmonary resuscitation; OR, odds ratio; ROSC, return of spontaneous circulation. ^b^If pre-hospital epinephrine was received, variables were divided into three categories according to the time from the start of CPR by emergency medical services personnel to the first epinephrine administration (epinephrine administration time).

## Discussion

The present analyses based on a large, population-based, nationwide database of Japanese patients who had experienced OHCA show that initial shockable rhythm is associated with both one-month survival and one-month favorable neurological outcomes and is considered a key variable for CPR. This finding is consistent with a previous meta-analytic study by Sasson *et al*. [5]. They conclusively affirmed the critical importance of shockable rhythm for outcomes, along with bystander CPR and ROSC, in the prehospital setting. On the basis of these results, we have further investigated the effectiveness of prehospital epinephrine administration for OHCA patients with each initial cardiac rhythm. In the initial shockable rhythm cohort, the ratios of both short-term and long-term outcomes in the nonepinephrine group were significantly higher than those in the epinephrine group. However, in the initial nonshockable rhythm cohort, the ratios of pre-hospital ROSC and one-month survival in the epinephrine group were significantly higher than those in the nonepinephrine group, and no significant difference between the epinephrine and nonepinephrine groups for one-month CPC score of 1 or 2 was found.

Although there were no beneficial effects of prehospital epinephrine administration on one-month outcomes in patients with initial shockable rhythms after OHCA, prehospital epinephrine administration for OHCA patients with nonshockable initial rhythms is independently associated with one-month survival, when the epinephrine administration time was less than 20 minutes. To the best of our knowledge, this study is the first to show that pre-hospital epinephrine administration significantly improves one-month survival after OHCA in patients with initial nonshockable rhythms associated with its administration time. Unlike previous observational studies [2,3] that were underpowered to show this crucial association, our study is sufficiently large to identify the important beneficial effect of epinephrine on one-month survival after OHCA with initial nonshockable rhythms. We have also demonstrated that prehospital epinephrine administration is independently associated with deterioration in neurological outcomes at one month after cardiac arrest with both initial shockable and nonshockable rhythms when the epinephrine administration time was 10 minutes or longer.

Epinephrine hydrochloride produces beneficial effects in patients during cardiac arrest, primarily because of its α-adrenergic receptor-stimulating properties [13,24]. The α-adrenergic effects of epinephrine can increase both coronary and cerebral perfusion pressures during CPR [13]. The value and safety of the β-adrenergic effects of epinephrine are controversial because they may increase myocardial work and reduce subendocardial perfusion [13]. Laboratory data suggest that harmful epinephrine-induced reductions in microvascular blood flow during and after CPR may offset the beneficial epinephrine-induced increase in arterial blood pressure during CPR [16-18]. However, epinephrine is the recommended first-line drug for the resuscitation of patients with both shockable and non-shockable initial rhythms [13].

A recent RCT by Olasveengen *et al*. [14] showed higher ratios of short-term survival (any ROSC during resuscitation, hospital admission or intensive care unit admission) in the drug administration cohort (epinephrine, 79%; atropine, 46%; amiodarone, 17%) than in the control cohort, but it failed to show improvements in long-term survival (hospital discharge and one year after cardiac arrest). In their subgroup analysis, nonshockable rhythm had threefold higher ratios for achievement of ROSC with intravenous treatment. Although there may be some confounding factors, the same tendency was found in our current study. The ROSC achievement ratio in our nonshockable rhythm cohort was sixfold higher with pre-hospital epinephrine treatment (*P *< 0.001) (Table 3). Multivariate logistic regression analysis in the nonshockable rhythm cohort clearly indicated the effectiveness of prehospital epinephrine for prehospital ROSC with a high adjusted OR (Table 5). However, unlike the report by Olasveengen *et al*., which showed no differences in outcomes between with and without intravenous treatment for patients with shockable rhythms, our present study indicates that shockable rhythms had significant differences in outcomes for administration of epinephrine (*P *< 0.001) (Table 3). Multivariate logistic regression analyses in our study revealed that epinephrine administration for shockable rhythms worsened neurological outcomes at one month (Table 4). As epinephrine did not have a deteriorative effect on one-month survival for shockable rhythms when epinephrine administration time was 9 minutes or less (Table 4), this harmful effect of epinephrine on neurological outcomes for initial shockable rhythms may be related to the administration time of epinephrine and indication bias for epinephrine administration after the shock delivery. Others have found detrimental effects of epinephrine in patients with VF [15,25]. Moreover, the majority of survivors are VF patients who respond to the first one or two defibrillations and hence have no need for subsequent drug administration during resuscitation. These patients show a higher survival rate than those who require drug intervention [26]. Therefore, comparisons of the effects of epinephrine on long-term survival after OHCA in patients with VF are likely to be biased, and it is difficult to determine whether epinephrine provides long-term benefits for such patients.

An RCT by Jacobs *et al*. [15] demonstrated that epinephrine resulted in a statistically significant increase in ROSC, but not in the primary outcome of survival to hospital discharge. Their study also suggested that short-term survival following epinephrine administration after OHCA differed by cardiac rhythm. The treatment effect of epinephrine on prehospital ROSC was more marked in patients with nonshockable rhythms than it was in patients with shockable rhythms. Their results for achievement of ROSC were consistent with our current results. Moreover, we have demonstrated that prehospital epinephrine administration for OHCA patients with initial nonshockable rhythms was independently associated with improved one-month survival when epinephrine administration time was less than 20 minutes (adjusted OR, 1.78, 1.29; 95% CI, 1.50 to 2.10, 1.17 to 1.43; for epinephrine administration times 9 minutes or less and 10 to 19 minutes, respectively) (Table 5).

Hagihara *et al*. [6] conducted an observational study with a nationwide database in Japan. Using a propensity score analysis, they indicated that prehospital epinephrine use may be associated with poorer one-month survival and worse neurologic outcomes at one month after cardiac arrest. Their results are inconsistent with our present results. Unlike our current study, they did not include the epinephrine administration time variable as an important key confounding factor. Moreover, although certified ELSTs in Japan have been permitted to administer intravenous epinephrine in the field under online physician instruction since April 2006, they selected the data from 2005 to 2008. This database selection may have bias for the indication of epinephrine. We have selected the data from 2009 to 2010 for our analysis under the Japanese CPR guidelines [21], which are based on the 2005 American Heart Association guidelines [4].

Another observational study in Osaka showed that prehospital epinephrine administration had no significant effect on one-month survival in bystander-witnessed nontraumatic OHCA adults with initial non-VF and/or non-ventricular tachycardia rhythms [3]. These results are also inconsistent with our present results. This difference may be due mainly to differences in study participants. From among 209,577 patients in the large nationwide database, 194,085 patients with initial nonshockable rhythms after OHCA including any cardiac arrest causes were evaluated in our study. On the other hand, they evaluated only selected 3,161 patients with witnessed nontraumatic OHCA from the Osaka Utstein registry database with exclusion of shock-responded VF and/or ventricular tachycardia patients. Of those cardiac arrest patients, 2,655 patients with initial nonshockable rhythms were studied using multivariate logistic regression analysis for nine variables. We have analyzed eligible data using multivariate logistic analysis for 11 variables to reduce known confounding factors.

Recently, Nakahara *et al*. [2] analyzed cases in a nationwide Japanese database recorded between 2007 and 2008 and reported that cardiac origin OHCA patients who received early epinephrine administration (epinephrine administration time 10 minutes or less) had significantly higher ratios of intact neurological survival. Because they analyzed only 49,165 (23.2%) of 212,088 adult OHCA patients for the study after excluding no witnessed arrest (59.2%), OHCA due to external causes and early ROSC without epinephrine administration, there would be some selection bias. Their results are inconsistent with our current results. We could not indicate the beneficial effect of prehospital epinephrine on neurological outcomes at one month in both shockable and nonshockable rhythms, even if the epinephrine administration time was less than 9 minutes. These differences may derive mainly from patient selection bias.

In our present results, initial PEA rhythm was a crucial independent factor for prehospital ROSC, one-month survival and one-month CPC score of 1 or 2 in the nonshockable rhythm cohort (Table 5). These results may reflect time-dependent effects of epinephrine administration in patients with cardiac arrest with PEA. Nordseth *et al*. investigated the time-dependent effects of epinephrine on clinical state transition in patients with initial PEA and found that epinephrine has notable clinical effects, including "speeding up" the rate of transition and extending the time window for ROSC development [27]. PEA is categorized into the following three clinical states: "normotensive PEA" with baseline cardiac contractions, "pseudo-PEA" with decreased cardiac contractions and "true-PEA" with no cardiac contractions [28]. Intravenous epinephrine seems appealing in the latter two categories to promote ROSC [27]. These effects of epinephrine may ultimately contribute to one-month survival. Arrich *et al*. [29] reported that total epinephrine dose during asystole and PEA cardiac arrests was associated with an unfavorable neurological outcome and increased in-hospital mortality. This implies that another drug combination or a new protocol is required if prehospital epinephrine is not effective in OHCA patients with initial nonshockable rhythms. Experimental data suggest that simultaneous administration of epinephrine and nitroglycerin or atenolol may lead to a better outcome than the administration of epinephrine alone [30,31]. However, there are no definitive data from human studies supporting this hypothesis.

### Study limitations

The potential limitations of the current analyses are as follows. First, the major limitation is that patients with prehospital epinephrine administration were not assigned by randomized selection. Because limited certified ELSTs have been permitted to administer intravenous epinephrine under online medical control in Japan, the EMS personnel organization or their individual skills may have influenced the current results. In our current study, epinephrine use was indicated only for nonshockable rhythm refractory to chest compression or shock delivery after shockable rhythm. This would tend to bias the epinephrine patients toward worse outcomes and diminish the ROSC, one-month survival and CPC score 1 or 2 improvements. Second, unmeasured confounding factors in our study might have influenced our results. We did not evaluate in detail the in-hospital treatments, such as induced hypothermia [32], extracorporeal CPR [33] and drugs other than epinephrine, which may have affected the results. We assumed that OHCA patients received standard advanced life support according to the Japanese CPR guidelines [21], which are based on the 2005 American Heart Association guidelines [4]. Additionally, we did not have sufficient data for patients with OHCA, such as underlying disease, the place where the cardiac arrest occurred and the quality of bystander CPR. Although the nationwide database has used the Utstein-style guidelines for reporting cardiac arrest, we had no such detailed data and could not include that data in our analyses. Third, we did not evaluate the relation between the total dosages of epinephrine and outcomes. Repeated dosages of epinephrine were administered upon the physician's instruction after the first epinephrine administration was refractory. This instruction itself may be influenced by a judgment of the physician in charge. Moreover, one important confounder in this analysis is that patients without prehospital epinephrine administration may have received epinephrine after arrival at the hospital. Total cumulative epinephrine dosage of 15 mg or higher has been reported to influence the outcomes of OHCA patients [34]. This is considered to be associated with impaired tissue oxygen utility and impaired lactate clearance in the postresuscitation period. However, we did not have the detailed cumulative dose of epinephrine, including in-hospital dosages, for each patient. Consequently, we could not analyze the administration dosages of epinephrine, mainly due to lack of sufficient data. Fourth, it is not known whether our results are applicable to other communities with different emergency care characteristics. It may be necessary for researchers in other countries to validate our results.

## Conclusions

Prehospital epinephrine administration for OHCA patients with initial nonshockable rhythms was independently associated with achievement of pre-hospital ROSC and was also associated with improved one-month survival when epinephrine administration time was less than 20 minutes.

## Key messages

• We analyzed nationwide Utstein-style Japanese data collected over two years and found that initial cardiac rhythm was a crucial prehospital factor for predicting both survival and favorable neurological outcomes at one month.

• In the initial shockable rhythm cohort, the ratios of pre-hospital ROSC, one-month survival and one-month CPC score of 1 or 2 in the nonepinephrine group were significantly higher than those in the epinephrine group. However, in the initial nonshockable rhythm cohort, the ratios of pre-hospital ROSC and one-month survival in the epinephrine group were significantly higher than those in the nonepinephrine group. No significant difference between the epinephrine and nonepinephrine groups for one-month CPC score of 1 or 2 was found in the initial nonshockable rhythm cohort.

• In OHCA patients with initial shockable rhythms, only prehospital epinephrine administration with an administration time less than 9 minutes was independently associated with increased odds of prehospital ROSC.

• Prehospital epinephrine administration for OHCA patients with initial nonshockable rhythms was independently associated with prehospital ROSC and had an association with improved one-month survival when epinephrine administration time was less than 20 minutes.

• Prehospital epinephrine administration for OHCA patients with initial nonshockable rhythms was independently associated with deteriorated neurological outcomes at one month when the epinephrine administration time was 10 minutes or more.

## Abbreviations

CI: Confidence interval; CPC: Cerebral Performance Category; CPR: Cardiopulmonary resuscitation; ELST: Emergency life-saving technician; EMS: Emergency medical services; FDMA: Fire and Disaster Management Agency; OHCA: Out-of-hospital cardiac arrests; OR: Odds ratio; PEA: Pulseless electrical activity; RCT: Randomized controlled trial; ROSC: Return of spontaneous circulation; VF: Ventricular fibrillation.

## Competing interests

The authors declare that they have no competing interests.

## Authors' contributions

YG and TM designed the study. YG, TM and YNG conducted data cleaning. YG and YNG analyzed the data. YG drafted the manuscript. YNG and TM contributed substantially to its revision. YG takes responsibility for the paper as a whole. All authors approved the manuscript before submission.
